# p21^CDKN1A^ Regulates the Binding of Poly(ADP-Ribose) Polymerase-1 to DNA Repair Intermediates

**DOI:** 10.1371/journal.pone.0146031

**Published:** 2016-01-05

**Authors:** Ilaria Dutto, Maria Sukhanova, Micol Tillhon, Ornella Cazzalini, Lucia A. Stivala, A. Ivana Scovassi, Olga Lavrik, Ennio Prosperi

**Affiliations:** 1 Istituto di Genetica Molecolare del CNR, Via Abbiategrasso, 207, Pavia, Italy; 2 Institute of Chemical Biology and Fundamental Medicine, Russian Academy of Sciences, Prospekt Lavrentiev 8, Novosibirsk, Russian Federation; 3 Dipartimento di Medicina Molecolare, Immunologia e Patologia, Università di Pavia, Via Ferrata 9, Pavia, Italy; Tel-Aviv University, ISRAEL

## Abstract

The cell cycle inhibitor p21^CDKN1A^ was previously found to interact directly with DNA nick-sensor poly(ADP-ribose) polymerase-1 (PARP-1) and to promote base excision repair (BER). However, the molecular mechanism responsible for this BER-related association of p21 with PARP-1 remains to be clarified. In this study we investigate the capability of p21 to influence PARP-1 binding to DNA repair intermediates in a reconstituted BER system *in vitro*. Using model photoreactive BER substrates containing single-strand breaks, we found that full-length recombinant GST-tagged p21 but not a C-terminal domain truncated form of p21 was able to stimulate the PARP-1 binding to BER intermediates with no significant influence on the catalytic activity of PARP-1. In addition, we investigate whether the activation of PARP-1 through poly(ADP-ribose) (PAR) synthesis, is required for its interaction with p21. We have found that in human fibroblasts and in HeLa cells treated with the DNA alkylating agent N-methyl-N'-nitro-N-nitrosoguanidine (MNNG), the interaction of p21 with PARP-1 was greatly dependent on PAR synthesis. In fact, an anti-PAR antibody was able to co-immunoprecipitate p21 and PARP-1 from extracts of MNNG-treated cells, while blocking PAR synthesis with the PARP-1 inhibitor Olaparib, drastically reduced the amount of p21 co-immunoprecipitated by a PARP-1 antibody. Our results provide the first evidence that p21 can stimulate the binding of PARP-1 to DNA repair intermediates, and that this cooperation requires PAR synthesis.

## Introduction

Poly(ADP-ribose) polymerase-1 (PARP-1) belongs to the PARP superfamily [1] and is involved in several pathways, such as DNA replication, DNA repair, cell death, transcription, mitochondrial activity regulation [2–9] and modulation of chromatin structure [10].

It is widely accepted that PARP-1 is capable of rapidly recognizing DNA damage in the form of single-strand breaks (SSBs) caused by genotoxic agents or DNA glycosylase and apurinic/apyrimidinic endonuclease 1 (APE1) during base excision repair (BER) [11–14].

Upon recognition of SSBs, PARP-1 activates and begins to synthesize poly(ADP-ribose) (PAR) [15], and to recruit BER factors, such as XRCC1, DNA polymerase (pol) ß and DNA ligase III [12,16,17], in order to promote DNA repair and prevent the conversion of SSBs to double-strand breaks [18] (which can also be recognized by PARP-1) [19–21].

Binding of PARP-1 to damaged DNA and its subsequent auto-PARylation are important steps in DNA repair response, especially in the BER process [22–25]. In fact, the BER process can be impaired by inhibiting PARP-1 and thus preventing PAR synthesis, which causes the stabilization of PARP-1 interaction with DNA lesions and impairs recruitment of BER factors [22,26–30]. However, the molecular basis of PARP-1 binding to DNA and *in situ* auto-modification are not fully elucidated as two different mechanisms have been suggested: the first involves an intra-molecular conformational change leading to an inter-domain interaction between the DNA binding region and the catalytic domain [31] while the second advocates for an auto-modification in trans caused by PARP-1 dimer formation [32].

The role of PARP-1 in DNA repair mechanisms is tightly regulated [33]. In this respect, the cell cycle inhibitor p21^CDKN1A^ plays an important role in regulating PARP-1. In fact, p21 is a key regulator of the DNA damage response at multiple levels: cell cycle, protein transcription, apoptosis and DNA repair [34–36]. The involvement of p21 in the BER process was initially suggested due to its ability to interact with BER factors, such as proliferating cell nuclear antigen (PCNA) and APE1 [37]. We found that the association of PARP-1 with PCNA resulted in the inhibition of DNA synthesis in vitro [38]. However, an interaction with chromatin-bound PCNA after DNA damage was detected only in replicating HeLa cells, but not in slowly proliferating or quiescent human fibroblasts [38,39]. A direct association between the C-terminal region of p21 and PARP-1 (causing the latter’s inhibition) independent of PCNA, was also determined [38]. In subsequent studies, p21 was found to interact with the auto-modification/DNA binding domains of PARP-1 [40]. The importance of this interaction in the BER process was supported by evidence that p21^−/−^ cells were more sensitive to alkylating agents and showed BER deficiency, possibly due to persistent DNA binding of PARP-1 and an abnormally high PAR production [40]. These findings suggested that p21 might be necessary to regulate PARP-1 activity for the correct turnover of BER factors at the DNA damaged sites [41]. However, it remained unclear how p21 is capable of fulfilling this function.

To better understand the mechanism by which p21 can influence the BER process, we have investigated whether the association between p21 and PARP-1 is dependent on PARP-1 interaction with damaged DNA and on the subsequent PARP-1 activation leading to PAR polymer synthesis. By using photoreactive BER intermediates synthesized *in situ* by pol ß [42], we have monitored the binding of PARP-1 to nicked DNA in the presence or absence of p21. Furthermore, by using the specific PARP inhibitor Olaparib [43], we have analysed the influence of PARP-1 activity on its interaction with p21 at the cell level. We have found that p21 stimulates the binding of PARP-1 to nicked DNA substrates mimicking BER intermediates and that recruitment of PARP-1 to chromatin after DNA damage is significantly reduced in p21^−/−^ human fibroblasts. In addition, we show that *in vivo* PAR synthesis facilitates the interaction of PARP-1 with p21, as Olaparib significantly reduced this association.

These results provide novel clues for the regulation of the BER process, as they indicate p21 as a new activator of the DNA damage recognition step of PARP-1, and further highlight the role of p21 as a cooperative factor for DNA repair.

## Materials and Methods

### Cell cultures, proteins and reagents

LF-1 human lung embryonic fibroblasts and hTERT-immortalized p21^+/+^ and p21^−/−^ fibroblasts derived from LF-1 cells were obtained from prof. J.M. Sedivy (Brown University, USA) and were previously described [44,45]. LF-1 fibroblasts were cultured in Minimum Essential Medium (MEM) supplemented with 10% (v/v) fetal bovine serum (FBS, Gibco). To obtain quiescent cultures, cells were grown to confluence and serum starved (0.5% FBS) for 3–5 days. Cultures of p21^+/+^ and p21^−/−^ fibroblasts were grown in HAM F-10 medium, as previously described [40]. HeLa S3 cells (ATCC) were grown in D-MEM supplemented with 10% FBS (Gibco), 0.1 mg/ml penicillin, 100 U/ml streptomycin, 2 mM glutamine and 2% (w/v) sodium pyruvate (EuroClone). N-methyl-N-nitro-N-nitroso guanidine (MNNG, Sigma) was prepared as 50 mM stock solution in DMSO. Olaparib (SelleckChem) was dissolved in DMSO (100 mM stock solution) and used at the final concentration of 10 μM.

[γ-^32^P]ATP, (4000 Ci/mmol) and [^32^P]NAD (600 Ci/mmol) were provided by the Laboratory of Biotechnology (ICBFM, Novosibirsk, Russia). Synthetic oligonucleotides were obtained from Laboratory of Medical Chemistry (ICBFM, Novosibirsk, Russia). The photoreactive dCTP analog, FABGdCTP (exo-N-[4-(4-azido-2,3,5,6,-tetrafluorobenzylidenehydrazinocarbonyl)-butylcarbamoyl]-2'-deoxycytidine-5'-triphosphate), was kindly gifted from Dr. I.V. Safronov (ICBFM, Novosibirsk, Russia).

Transfection of HeLa cells with selected plasmids was performed with Effectene reagent (Qiagen). GST-tagged constructs coding for full-length (FL) PARP-1, the N-terminal region encompassing the DNA binding and auto-modification domain (A-D) and the C-terminal region containing the catalytic domain (E-F) were obtained from Josiane and Gilbert de Murcia (CNRS-ESBS, Illkirch, France), as previously described [40]. The vector used for the expression of p21 fused to GFP (p21-GFP) has also been previously described [46]. Recombinant GST-tagged human p21 full length (p21FL), or a truncated form containing the C-terminal domain (p21Cter), were expressed in bacteria and purified, as previously reported [47].

The plasmids bearing cDNAs of human PARP-1 were kindly provided by Dr. M. Satoh (Laval University, Canada), the expression vectors for rat pol ß and human APE1 were a generous gift of Dr. S. H. Wilson (NIEHS, NIH, USA). The recombinant proteins PARP-1, APE1 and pol ß were overexpressed in *E*. *coli* and purified, as previously described [48].

T4 polynucleotide kinase and recombinant *E*. *coli* Uracil DNA glycosylase (UDG) were provided by Biosan (Novosibirsk, Russia). Bovine testis nuclear extract (BTNE) was prepared as previously described [42]. Protein concentrations were measured by Bradford assay.

Rabbit polyclonal anti-p21 (C-19 and N-20), and anti-XRCC1 (N-15) antibodies were from Santa Cruz. Mouse monoclonal antibodies against actin (AC40), p21 (CP74), and the polyclonal anti-GST antibody were from Sigma. Monoclonal antibodies against PARP-1 (F1-23), and PAR (10H) were from Alexis. Mouse monoclonal antibody against PCNA (PC10) was from DakoCytomation. Polyclonal antibody anti-pol ß was from Novus Biologicals. Polyclonal antibody to PAR was from Millipore. Rabbit anti-GFP polyclonal antibody, and goat anti-mouse and anti-rabbit IgG secondary antibodies conjugated with Alexa 488, or Alexa 594, were from Molecular Probes (Life Technologies).

### DNA substrates and 5'-end labeling

Oligodeoxyribonucleotides were 5'-^32^P-phosphorylated with T4 polynucleotide kinase and purified by 20% polyacrylamide/7.0 M urea gel electrophoresis as described [42], followed by electro-elution and precipitation with 2% solution of LiClO_4_ in acetone. The precipitated oligodeoxyribonucleotides were dissolved in 10 mM Tris-HCl (pH 8.0) and 1 mM EDTA (TE buffer). Complementary oligonucleotides were annealed by heating (90°C, 5 min) a solution of equimolar amounts, followed by slow cooling-down to room temperature, to form the double-stranded DNA substrates containing nick (DNApF and DNAp), uracil (DNA-U) or 3-hydroxy-2-hydroxymethyltetrahydrofuran (DNA-F). The structures of DNA duplexes used are summarized in Table 1.

**Table 1:** Nucleotide sequences, designations and structures of DNA substrates used for photocrosslinking and assay for PARP1 autoPARylation.

Nucleotide sequence and structure	Designation
5'-[^32^P]-GGCTTCATCGTTGTC**F**CAGACCTGGTGGATACCG-3'	DNA-F
3'-CCGAAGTAGCAACAG**G**GTCTGGACCACCTATGGC-5'	
5'-[^32^P]-GGCTTCATCGTTGTC**U**CAGACCTGGTGGATACCG-3'	DNA-U
3'-CCG AAGTAGCAACAG**G**GTCTGGACCACCTATGGC-5	
5'-[^32^P]-GGCTTCATCGTTGTC**C**^**FABG**^pF*CAGACCTGGTGGATACCG-3'	DNA*pF
3'-CCG AAGTAGCAACAG**G**GTCTGGACCACCTATGGC-5'	
5'-[^32^P]-GGCTTCATCGTTGTC**C**^**FABG**^p**CAGACCTGGTGGATACCG-3'	DNA*p
3'-CCGAAGTAGCAACAG**G**GTCTGGACCACCTATGGC-5'	
5'-[^32^P]-GGCTTCATCGTTGTCCp**CAGACCTGGTGGATACCG-3'	DNAp
3'-CCGAAGTAGCAACAG**G**GTCTGGACCACCTATGGC-5'	
5'-[^32^P]-GGCTTCATCGTTGTCCpF*CAGACCTGGTGGATACCG-3'	DNApF
3'-CCGAAGTAGCAACAG**G**GTCTGGACCACCTATGGC-5'	

Abbreviations

**U-**uracil

**F-**3-hydroxy-2-hydroxymethyltetrahydrofuran

pF* = 3-hydroxy-2-hydroxymethyltetrahydrofuran with 5'-phosphate

p** = phosphate

^**FABG**^**C**, exo-N-[4-(4-azido-2,3,5,6,-tetrafluorobenzylidenehydrazinocarbonyl)-butylcarbamoyl]-2'-deoxycytidine-5'-monophosphate.

### Preparation of photoreactive nicked DNA substrates

To generate photoactive DNA*pF or DNA*p (Table 1), the standard reaction mixtures (300 μl) contained 50 mM Tris-HCl (pH 8.0), 50 mM NaCl, 10 mM MgCl_2_, 0.1 μM pol ß and 70 μM FABGdCTP, 0.8 μM 5'-[^32^P]- labeled DNA containing one nucleotide gap with 5’-phosphate or 5’-furanophosphate. The one nucleotide gapped DNA substrates were generated by treating DNA-F with 20 nM APE1, DNA-U with 20 nM UDG and 20 nM APE1 before the reaction. The reaction mixtures were incubated for 15 min at 37°C to allow pol ß–dependent elongation of the primers with FABGdCMP and the reaction was then stopped by adding EDTA to a 20 mM final concentration, followed by precipitation by adding 1/10 volume of 3 M Na Acetate (pH 5.0), and 2.5 volumes of 96% ethanol. The photoreactive DNAs (DNA*pF or DNA*p) were dissolved in TE buffer at a final concentration of 2 μM.

### Photocrosslinking assay

Crosslinking of PARP-1 was carried out in reaction mixtures (20 μl) that contained 40 mM Tris-HCl (pH 7.5), 40 mM NaCl, 10 mM MgCl_2_, 0.1 μM (DNA*pF or DNA*p) and 0.1 μM human recombinant PARP-1 or BTNE (1.25 mg/ml). To these mixtures, 0.5 mM NAD^+^, 1.2 μM p21 or p21Cter were added, as indicated in the figure legends. The reaction mixtures were incubated at 37°C for 3 min then irradiated by UV light (λ = 312 nm, 1.5 J/cm^2^) on ice. A Bio-Link BLX-312 cross-linker (Vilber-Lourmat) was used as light source in all experiments. Reactions were stopped by adding SDS-sample buffer and heating for 5 min at 96°C. Photocrosslinking products were separated in a 10% SDS**-**PAGE. The gels were dried and subjected to phosphor imaging for quantification using Molecular Imager/Quantity One software (Bio-Rad, USA) or “Typhoon” (Amersham Pharmacia Biotech, USA).

### PARP-1 poly(ADP-ribosyl)ation assay

The reaction mixture (60 μl) for auto-poly(ADP-ribosyl)ation of PARP-1 contained 20 mM Tris-HCl (pH 8.0), 20 mM NaCl, 1 mM DTT, 10 mM MgCl_2_, 0.2 μM nicked DNAp or DNApF (Table 1), 100 μM NAD^+^ + 0.5 μCi [^32^P]-NAD^+^, 0.1 mg/ml BSA, 60 nM PARP-1. p21 (1.2 μM) was added as indicated in the figure legends. The reaction was stopped at 5, 10, 20 or 30 min, by dropping aliquots of the mixture (6 μl) on 10% (v/v) trichloroacetic acid (TCA)-saturated 1 MM Whatman filter paper. Unincorporated [^32^P]-NAD^+^ was removed with three 5% TCA washes of 15 min, then the TCA was removed by 96% ethanol and the filters were dried and counted by Cherenkov.

### Immunofluorescence experiments

Human primary fibroblasts (LF-1) and HeLa cells, transfected with GST-PARP-1 and p21-GFP, were seeded on coverslips and treated, or not (controls) for 4 h with 10 μM Olaparib. Cells were then treated for 30 min with 25 μM MNNG and re-incubated in whole medium. Cells were then washed in PBS, lysed *in situ* in ice-cold buffer (10 mM Tris-HCl, pH 8.0, 2.5 mM MgCl_2_, 0.1% (v/v) Igepal, 10 mM ß-glycerophosphate, 0.2 mM PMSF and 0.1 mM Na_3_VO_4_) and finally washed in buffer containing 10 mM Tris-HCl (pH 8.0), 2.5 mM MgCl_2_, 10 mM ß-glycerophosphate, 0.2 mM PMSF and 0.1 mM Na_3_VO_4_ [49]. Thereafter, cells were fixed in 2% (v/v) formaldehyde for 5 min at room temperature (r.t.) and then post-fixed in 70% (v/v) ethanol at -20°C. Samples were blocked in PBS containing 0.2% (v/v) Tween 20 and 1% (w/v) BSA and then incubated with anti-GST rabbit polyclonal diluted 1:100 and anti-PAR (mouse monoclonal, 10H, 1:100) antibodies, for 1 h. After three washings, coverslips were incubated for 30 min with goat anti-rabbit or goat anti-mouse polyclonal antibodies (1:200) labelled with Alexa 594 (Molecular Probes). After immunoreaction, cells were stained with Hoechst 33258 dye (0.5 μg/ml) for 2 min at r.t. and then washed in PBS. Slides were mounted in Mowiol (Calbiochem) containing 0.25% 1,4-diazabicyclo-[2,2,2]-octane (Aldrich) as antifading agent and viewed with a BX51 Olympus microscope.

### Immunoprecipitation and pull-down assays

Human primary fibroblasts (LF-1) were treated for 30 min with 25 μM MNNG and then washed in PBS and re-incubated for 15 min in whole medium at 37°C. Nuclei were isolated with a buffer containing 10 mM Tris-HCl (pH 8.0), 2.5 mM MgCl_2_, 10 mM KCl, 0.25 M sucrose, 0.5 mM DTT, 0.5% Igepal, 10 mM ß-glycerophosphate, protease and phosphatase inhibitor cocktails. Nuclear proteins were then extracted with RIPA buffer containing 50 mM Tris-HCl (pH 8.0), 150 mM NaCl, 0.1% SDS, 0.5% sodium deoxycholate, 1% Igepal, protease and phosphatase inhibitor cocktails [50]. After centrifugation at 16,100 *g*, the soluble fraction was incubated (3 h, 4°C) with polyclonal anti-PAR or monoclonal anti-PARP-1 (F1-23) antibodies pre-bound to protein-G magnetic beads (Dynabeads, Life Technologies). Beads were then pelleted with a magnet and washed three times in 10 mM Tris-HCl buffer (pH 7.4) containing 150 mM NaCl, 1 mM PMSF, 10 mM sodium butyrate, 10 mM ß-glycerophosphate, protease and phosphatase inhibitor cocktails, to eliminate unspecific binding.

HeLa cells were transfected with PARP-1 fragments corresponding to N-terminal, C-terminal and full-length protein, fused with GST tag [40]. Cells were then treated, or not, with 10 μM Olaparib, and after 4 h, they were treated with 25 μM MNNG to induce DNA damage. Samples were lysed and an equal amount (1 mg) of each cell extracts was incubated with GSH-agarose beads (Qiagen) in binding buffer (50 mM Tris-HCl, pH 8.0, 2.5 mM MgCl_2_, 75 mM KCl, 5 mM DTT, 1 mM PMSF), under constant agitation for 1 hour at 4°C. After three washings in 50 mM Tris-HCl buffer (pH 8.0) containing 150 mM NaCl, 1 mM DTT, 1 mM PMSF, 0.5% Igepal), the beads were suspended in SDS-sample buffer and heated at 65°C for 10 min.

### Chromatin fractionation

To detect chromatin-bound PARP-1 in the presence of Olaparib, human p21^+/+^ and p21^−/−^ fibroblasts, cells were first exposed to MNNG as indicated previously and then collected at the end of treatment (t 0) or re-incubated in the presence or in the absence of Olaparib for further 30 min. Finally, cells were separated in soluble and chromatin-bound fractions and proteins in the latter fraction were released by DNase I digestion [49].

### Western blot analysis

Samples obtained by immunoprecipitation and pull-down experiments described previously were separated by SDS-PAGE on a 4–12% gradient gel (NuPage), transferred to nitrocellulose membrane and analysed by immunoblotting with the following antibodies: anti-PARP-1 (F1-23, 1:1000), anti-GST (1:3000), anti-XRCC1 (1:500), anti-pol ß (1:1000) and anti-p21 (1:1000). Thereafter, the membranes were incubated with specific HRP-conjugated secondary antibodies (Dako 1:2000) for 30 min at r.t. and immunoreactive bands were revealed by using ECL (Amersham, GE-HealthCare).

## Results

### p21 stimulates PARP-1 binding to DNA BER intermediates *in vitro*

Previous studies have shown that p21 and PARP-1 can cooperate in the regulation of BER [40], and that PARP-1 can be selectively photocrosslinked by BER intermediates containing a nick in mouse embryonic fibroblast (MEF), and in bovine testis nuclear extracts (BTNE) [12,42,51]. In this study, we have applied the photocrosslinking assay to investigate the influence of p21 on the interaction of PARP-1 with DNA repair intermediates in cell extracts. The photoreactive substrates were pre-synthesized by pol ß via incorporation of the FABGdCMP moiety onto the 3’-end of upstream primer and contained a nick with a 3-hydroxy-2-hydroxymethyltetrahydrofuran with 5-phosphate (5’-pF) (DNA*pF) or a 5-phosphate group (DNA*p) (Table 1). Given that the 5’-pF group cannot be removed by the lyase activity of pol ß [51], the DNA*pF can be considered as the central intermediate of BER at the stage just prior to 5′-deoxyribose phosphate lyase activity of pol ß in the short patch BER, or as an early intermediate in the long patch BER. The DNA*p may be considered as the penultimate product in the BER pathways [42].

**Fig 1:**
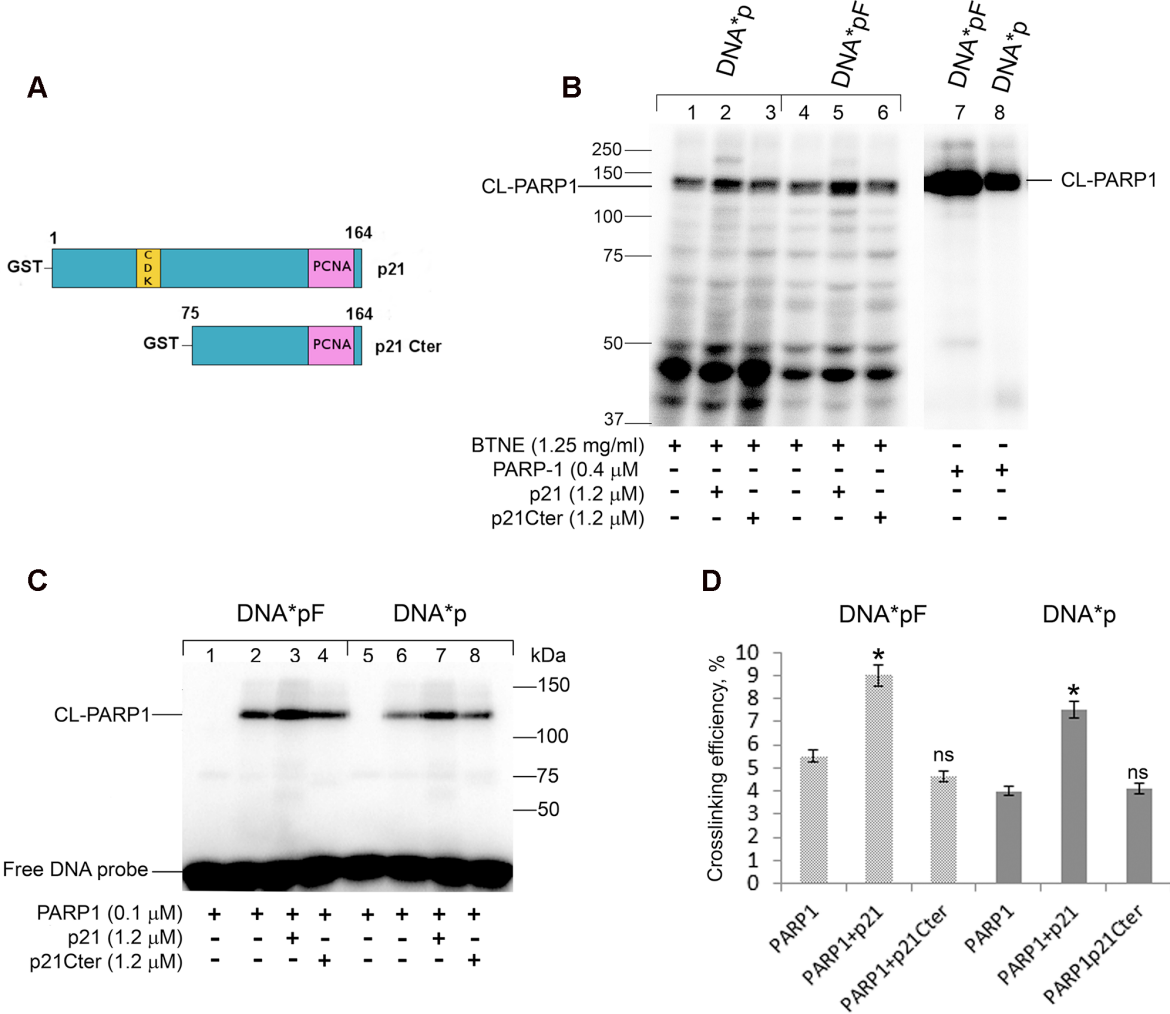
p21 increases the level of PARP-1 crosslinking to nick-containing BER DNA intermediates. (**A**) Schematic diagram showing the structure of p21 full length, with CDK and PCNA binding domains, and of the C terminal fragment (p21 Cter) lacking the first N-terminal 74 aa. (**B**) Crosslinking of PARP1 of the nuclear extract in the presence of p21 or p21Cter. [^32^P]-labeled 0.1 μM DNA*pF (lanes 1–3, 7) or DNA*p (lanes 4–6, 8) was incubated with bovine testis nuclear extract (BTNE) proteins (lanes 1–3, 4–6) in the presence of 1.2 μM p21 (lanes 2, 5) or p21Cter (lanes 3, 6). Loading control: DNA*pF, and DNA*p incubated with recombinant PARP-1 (lanes 7, 8). (**C**) Crosslinking of recombinant PARP1 in the presence of p21 or p21Cter. [^32^P]-labeled 0.1 μM DNA*pF (lanes 1–4) or DNA*p (lanes 5–8) was incubated with recombinant PARP-1 in the presence of p21 (lanes 3, 7) or p21Cter (lanes 4, 8). Then the mixtures were UV irradiated (lanes 2–4 and 6–8). Free DNA probe is also shown. The products were separated on 10% SDS-PAGE and analyzed by PhosphorImaging. The reaction conditions and analysis of the reaction products are described in “Materials and Methods”. The nucleotide sequence and structure of (DNA*pF and DNA*p) is reported (Table 1). (**D**) Diagram showing the quantitative analysis results shown in panel C. The crosslinking of PARP-1 was defined as the percentage (%) of photoreactive DNA, which formed covalent adducts with PARP-1. Error bars represent relative mean ± SD, *n* = 4. Asterisks denote statistically significant difference (*, *P*<0.05, ns, not significant; t-test).

First, we investigated whether p21 could influence PARP-1 crosslinking to nicked DNA duplexes in BTNE. To this purpose, the photoreactive DNA*pF or DNA*p were incubated with BTNE proteins in the presence of p21 full-length (p21), or a truncated form lacking the first N-terminal 74 amino acids (p21Cter) (Fig 1A), and then exposed to UV light to induce crosslinking (Fig 1B). Addition of p21 to BTNE resulted in a noticeable increase in the level of endogenous PARP-1 crosslinking to both DNA*pF and DNA*p (Fig 1B, lanes 2 and 5). In contrast to p21, addition of p21Cter did not influence the labelling of PARP-1 with the DNA substrates (Fig 1B, lanes 3 and 6). An even more pronounced effect of p21 on labelling of PARP-1 was observed for purified PARP-1 protein (Fig 1C and 1D). P21 significantly increased the crosslinking of purified PARP-1 to nicked DNA*pF, as well as to DNA*p, by more than 50% (*P*<0.05) (Fig 1D). These results indicate that p21 can influence the PARP-1 binding to nicked BER intermediates *in vitro*. In addition, this effect was more pronounced on the DNA substrate containing a nick with the 5’-pF group. On the whole, the photocrosslinking assay provides evidence that p21 stimulates the binding of PARP-1 to DNA nicks.

**Fig 2:**
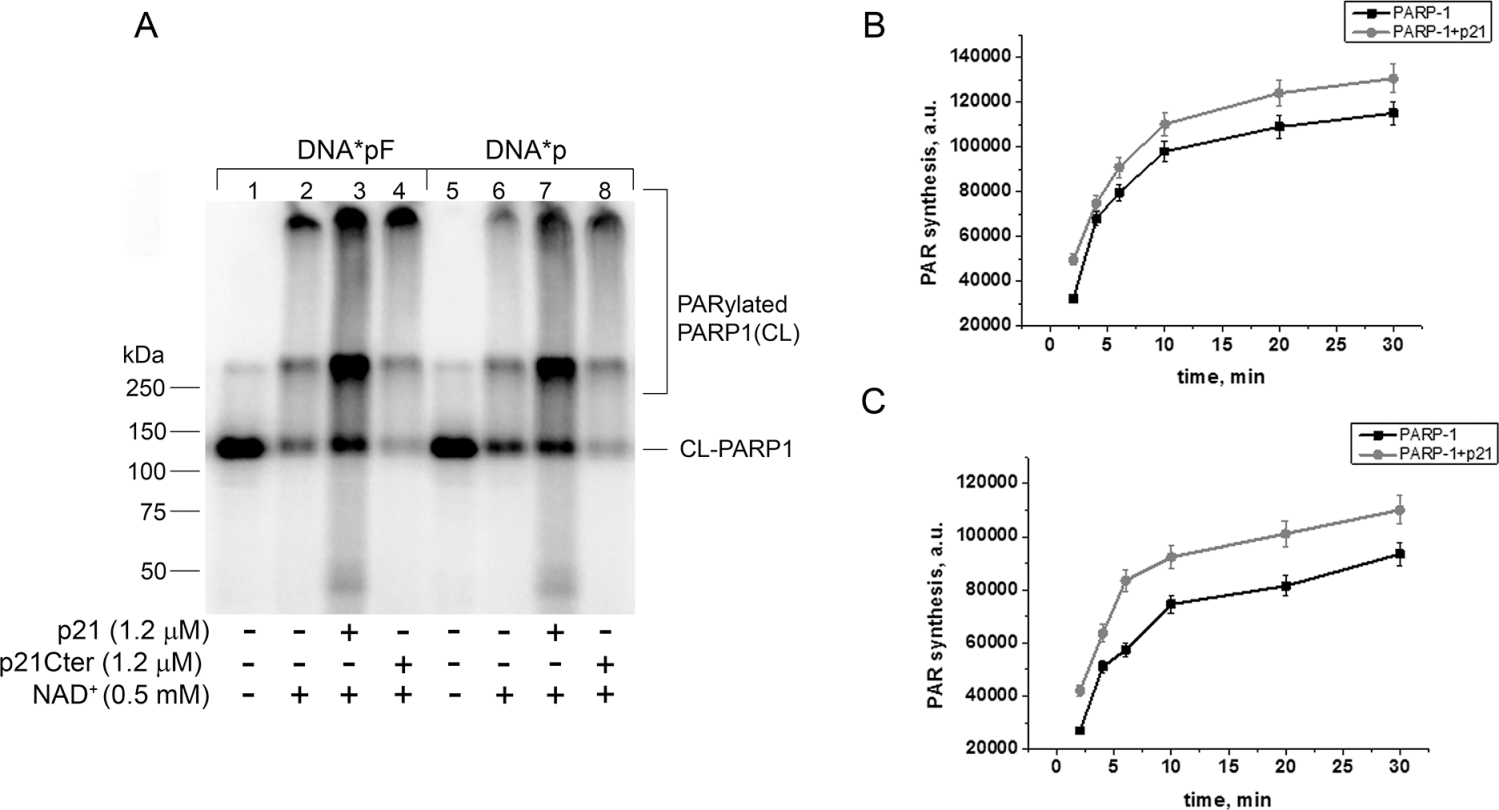
p21 stimulates the poly(ADP-ribosyl)ation activity of PARP-1 in the presence of nicked DNA. (**A**) Crosslinking assay of PARP1 activation in the presence (+) or absence (−) of p21. Photoreactive 5’-[^32^P]-labeled 0.1 μM DNA*pF (lanes 1–4) or DNA*p (lanes 5–8) were incubated with 0.1 μM PARP1 with (+) or without (−) NAD^+^. (**B,C**) Kinetic analysis of PARP-1 activity in the presence of p21. 0.2 μM nicked DNApF (B), or DNAp (C) was incubated with 60 nM PARP-1, 100 μM NAD^+^ + 0.5 μCi [^32^P]-NAD^+^ in the presence of 1.2 μM p21 (where indicated). The data are mean values ± S.D., *n* = 3.

Since the above experiments were performed in the absence of the PARP-1 substrate NAD^+^ (*i*.*e*. without PARP-1 catalytic activation), we also tested the effect of p21 on PARP-1 activity on nicked photoreactive DNA in the presence of NAD^+^. As expected, when the incubation mixture was supplemented with NAD^+^ before UV-light irradiation, PARP-1 labelling was reduced with a concomitant appearance of crosslinking products with slower electrophoretic mobility, and corresponding to labelling of PARylated PARP-1 (Fig 2A, lanes 2 and 6). When p21 was added to the reaction, the labelling of PARylated PARP-1 increased (Fig 2A, lanes 3 and 7), while the effect was not seen with p21Cter (Fig 2A, lanes 4 and 8). By monitoring the influence of p21 on the kinetics of PAR synthesis, in the presence of nicked DNA (DNApF or DNAp) and [^32^P]-labelled NAD^+^, we observed that p21 had only a modest or no effect on PARP-1 catalytic activity (Fig 2B and 2C, respectively). Cumulatively, these results indicate that *in vitro* p21 can modulate PARP-1 binding to DNA BER intermediates, without a net influence on the following PAR synthesis.

### Interaction of p21 with PARP-1 is mediated by PAR

**Fig 3:**
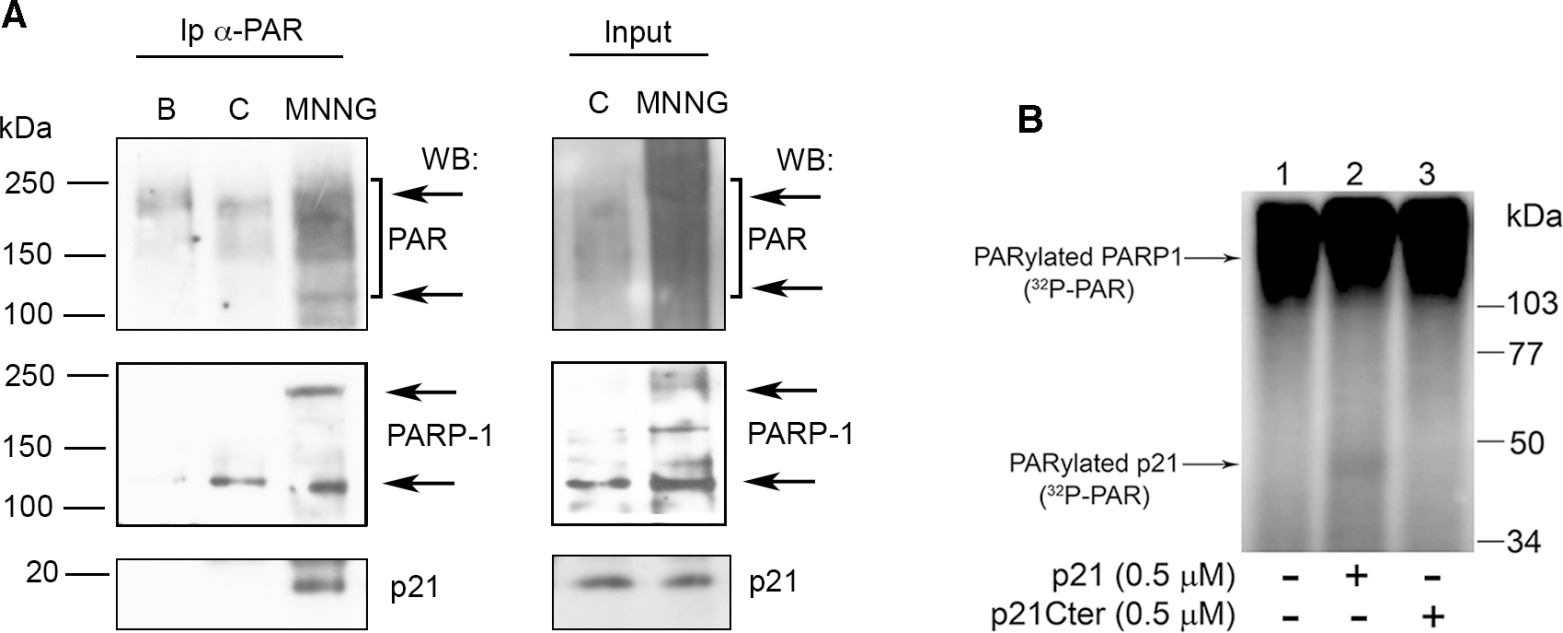
Interaction of p21 with PAR *in vivo*. (**A**) Immunoprecipitation (Ip) of p21 with polyclonal anti-PAR antibody from soluble (B), or nuclear extracts of LF-1 human fibroblasts untreated controls (C), or cells treated with 25 μM MNNG (MNNG). Input shows 10% of nuclear extracts before Ip. The samples were separated by SDS-PAGE and analysed by Western blot for PAR (10H antibody), PARP-1 (F1-23 antibody), and p21 (CP74 antibody). The arrows indicate the position of unmodified (upper) and PARylated (lower) forms of PARP-1. (**B**) PARylation of p21. Purified PARP1 (70 nM) was incubated without (lane 1, −), or with 500 nM p21 (lane 2, +) or p21C-term (lane 3, +) in the presence of [^32^P]-NAD^+^ and 0.1 A_260_/ml of DNase I-activated calf thymus DNA for 15 min at 37°C. Samples were analysed by 10% SDS-PAGE followed by phosphorimaging.

Next, we wanted to determine whether *in vivo* PAR chains formed during BER-dependent SSB production mediated the interaction of p21 with PARP-1. To this purpose, we performed immunoprecipitation with an antibody recognizing PAR polymer in cell extracts obtained from quiescent human fibroblasts, untreated or treated for 30 min with the DNA alkylating agent MNNG. The results showed that in the sample exposed to MNNG, the antibody reacted with PAR polymers corresponding mainly to auto-PARylated PARP-1, detectable as a higher molecular weight band (about 250 kDa) compared to the canonical unmodified form (about 113 kDa) (Fig 3A). Interestingly, in the sample immunoprecipitated from MNNG-treated cells, a band reacting with a p21-specific antibody was also detected. This result prompted us to investigate whether p21 itself may be a PARP-1 substrate, rather than only binding to PAR, as has been suggested in the past [52]. Incubation of recombinant GST-tagged p21 with purified PARP-1 showed that PARylated p21 was barely detectable (Fig 3B, lane 2).

**Fig 4:**
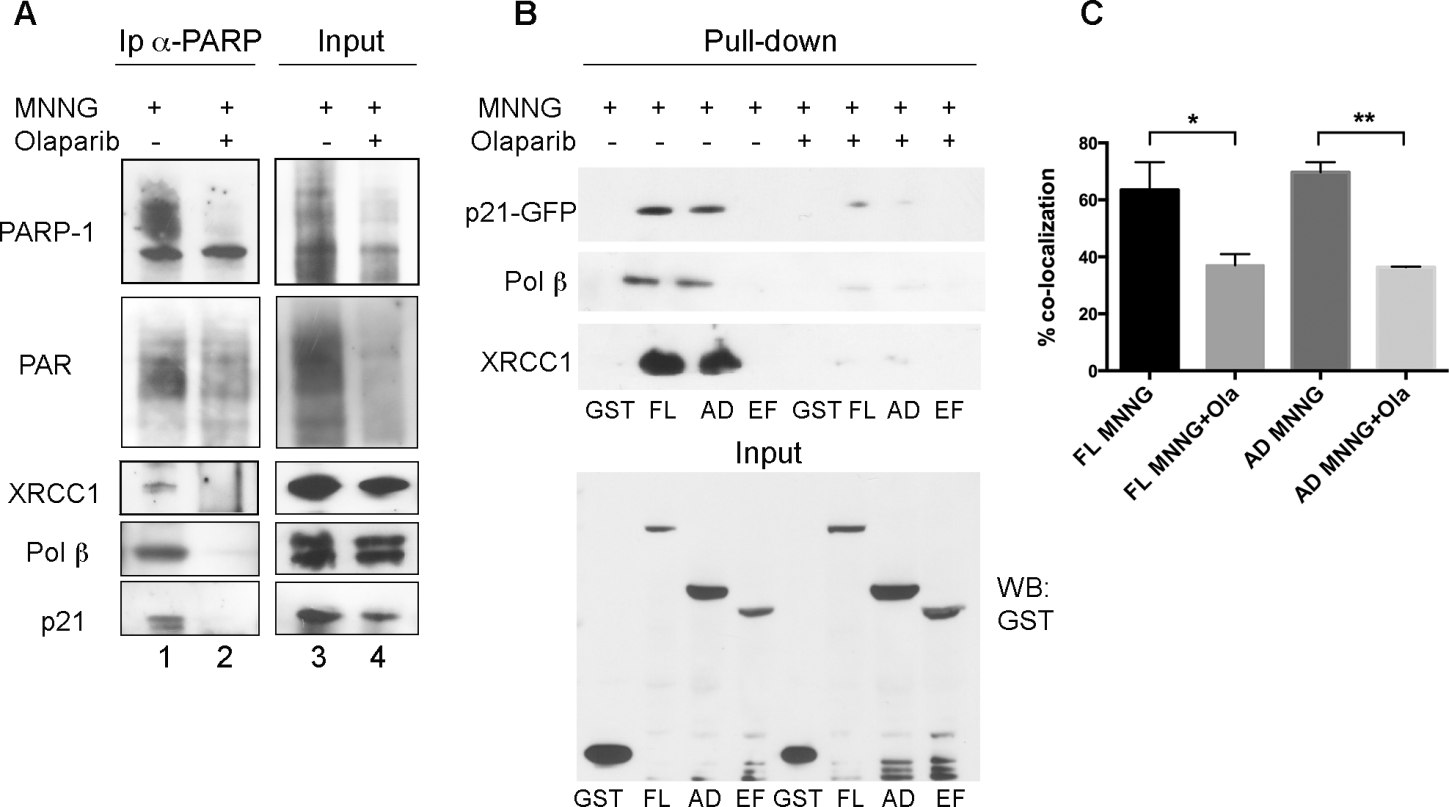
Interaction of p21 with PARP-1 is reduced by Olaparib. (**A**) Immunoprecipitation of p21 with monoclonal anti-PARP-1 F1-23 antibody from nuclear extracts of LF-1 human fibroblasts treated for 30 min with 25 μM MNNG alone (MNNG), or pre-treated with 10 μM Olaparib. Immunoprecipitates were separated by SDS-PAGE and analysed by Western blot for PARP-1, XRCC1, pol ß, or p21. Input shows 10% of total extract before Ip. (**B**) Pull-down of GST-tagged PARP-1 FL, AD, or EF fragments from HeLa cell extracts treated for 30 min with 25 μM MNNG in the presence, or in the absence of 10 μM Olaparib. Samples were analysed by Western blot for XRCC1, pol ß, or p21. Input shows 10% of total extract before pull-down. (**C**) Percentage of co-localization of GST-tagged PARP-1 FL, or AD constructs with p21-GFP in HeLa cells after treatment with 100 μM MNNG, in the presence or in the absence of 10 μM Olaparib. Chromatin-bound fractions of *in situ* lysed cells were analysed. Mean values ± S.D. of three independent experiments are shown.

To better characterize the interaction between p21 and PARP-1 mediated by DNA damage-induced PAR synthesis, human fibroblasts were pre-incubated with the PARP-1 inhibitor Olaparib, before treatment with MNNG. Under these conditions, Olaparib blocked MNNG-induced PAR synthesis (Fig 4A, lane 4), as also observed by immunofluorescence staining of PAR polymers with anti-PAR antibody (S1 Fig). Cells were collected and lysed for immunoprecipitation with anti-PARP-1 antibody, followed by Western blot analysis of PARP-1, XRCC1, pol ß, p21 proteins and PAR levels in each immunoprecipitate. Olaparib-mediated inhibition of PARP-1 not only prevented its association with p21, but also significantly reduced PARP-1 interaction with other important BER factors, such as XRCC1 and pol ß (Fig 4A, lane 2). To confirm these results, HeLa cells were co-transfected with vectors driving the expression of GST-tagged full-length PARP-1 (FL), the auto-modification and DNA-binding domain (AD), or the catalytic domain (EF), together with p21-GFP [40]. After affinity pull-down with GSH-agarose beads, the interaction between tagged PARP-1 proteins and p21-GFP was analysed by Western blot. The results, confirming the association of p21-GFP with the GST-PARP-1 FL or the AD domain [40], showed that their interaction was greatly abolished by the Olaparib-mediated inhibition of PARP-1 activity (Fig 4B). In agreement with the results obtained on fibroblasts, the interaction of PARP-1 with pol ß, and with XRCC1, was significantly reduced in the presence of Olaparib. To further confirm these results, the co-localization of p21 with chromatin-bound PARP-1 was investigated by immunofluorescence staining of exogenous PARP-1 FL, or the AD domain, with an antibody against the GST tag and by detecting p21-GFP autofluorescence (S2 Fig). In cells exposed to Olaparib before and during treatment with MNNG, the extent of co-localization was significantly reduced by about 50%, as compared with that observed in cells treated only with MNNG (Fig 4C).

### Absence of p21 affects PARP-1 recruitment to DNA damage

**Fig 5:**
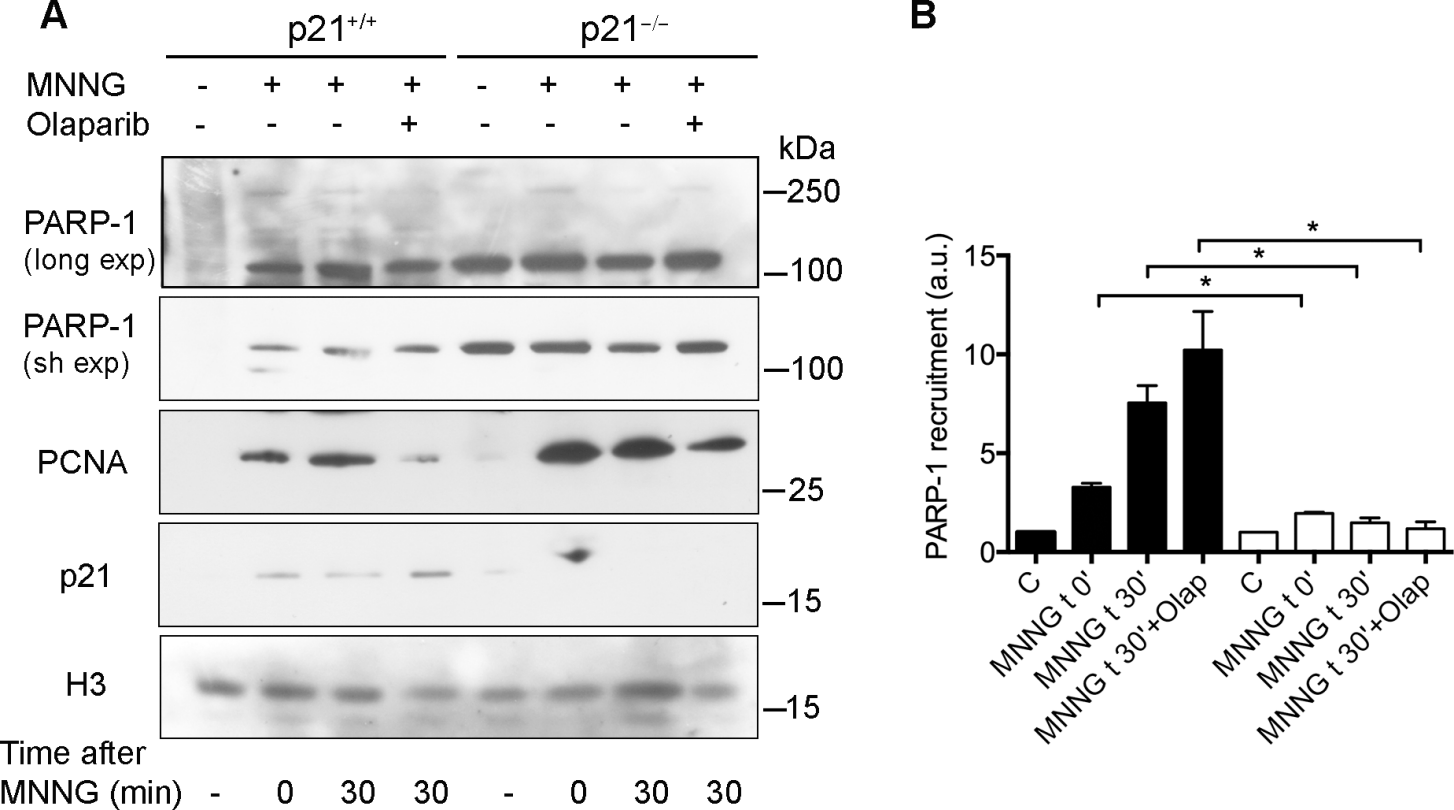
Reduced chromatin-binding of PARP-1 after DNA damage in p21^−/−^ fibroblasts. (**A**) Human p21^+/+^ and p21^−/−^ fibroblasts were treated with 25 μM MNNG and then collected immediately (t 0), or after 30 min recovery (t 30), in the absence or in the presence of 10 μM Olaparib. Chromatin-bound fractions were extracted and analysed by Western blot for PARP-1 after long and short (sh) film exposure (exp), and for PCNA, p21 or histone H3 (loading control). (**B**) Densitometric analysis of PARP-1 recruitment, compared to the untreated samples, after normalization to histone H3 content. Results are mean values ± S.D. of three independent experiments (*, *P*<0.05, t-test).

In order to obtain further insight into the requirement of p21 for PARP-1 binding to DNA after damage, both normal p21^+/+^ and p21^−/−^ human fibroblasts were exposed for 30 min to MNNG and then immediately collected or incubated in drug-free medium for further 30 min, both in the presence or absence of Olaparib. The chromatin-bound fraction of each sample was then isolated and samples were analysed by Western blot for PARP-1 binding to DNA lesion sites. In p21^+/+^ cells, PARP-1 was recruited to chromatin after DNA damage by MNNG in a time-dependent manner, and bands with slower mobility, relative to PARylated PARP-1, were detected only after long exposure of the blot (Fig 5A, long exp). In the presence of Olaparib, the high MW bands were less detectable (Fig 5A). In contrast, elevated levels of 113 kDa band representing PARP-1 were already observed in the untreated control p21^−/−^ cells, and further recruitment to chromatin after DNA damage was considerably limited, as compared with p21^+/+^ cells. High MW bands could also be detected in p21^−/−^ (Fig 5A, long exp), but no clear difference could be observed when compared to p21^+/+^ cells. Densitometric analysis of Western blot bands revealed that the amount of chromatin-bound PARP-1 (113 kDa band) increased by about 3 times immediately after MNNG treatment. During the post-treatment period, PARP-1 levels further increased in the presence of Olaparib, when compared to the levels observed in its absence in p21^+/+^ cells but not in p21^−/−^ cells (Fig 5B).

## Discussion

In the search for PARP-1 partners, we have previously demonstrated the physical association between p21 and the DNA binding domain of PARP-1 [40]. In order to investigate the possible modulation of the basic reactions of PARP-1 by p21 during DNA damage, in the present study we have carried out *in vitro* experiments using model DNA substrates mimicking BER intermediates which have been previously demonstrated to interact with PARP-1 in MEF extracts [42,53]. We have found that p21 increases the binding of PARP-1 to these BER intermediates, suggesting that p21 could exert a positive modulation of DNA binding by PARP-1 (Fig 1). This is an original observation, supported also by *in vivo* experiments, which confirmed that the interaction was not restricted to the purified enzyme, but could occur with the endogenous protein as well (Fig 1B).

We then addressed the possible modulation of PARP-1 catalytic activity and observed that p21 does not significantly influence the synthesis of PAR. This is in apparent contrast with the data we reported using a p21 C-terminal peptide, which was able to inhibit PARP-1 auto-modification on activated DNA [38]. However, the present evidence indicating that the full-length p21 protein does not have such an effect could be ascribed to the type of DNA substrate used (Fig 2). In fact, PARP-1 activity on DNA duplexes containing an abasic site, or a nick introduced by APE1 endonuclease, was considerably more efficient than on activated DNA, suggesting that PARP-1 prefers to bind to BER intermediates rather than SSBs.

To further investigate the impact of PAR synthesis on the p21-PARP-1 axis, we performed immunoprecipitation experiments with cell extracts from samples treated with the PARP inhibitor Olaparib. Given that under these conditions the inhibition of PARP-1 activity by Olaparib significantly reduced the association and the co-localization between the two proteins, we can assume that they may interact through PAR. This observation is in agreement with findings indicating that p21 recognizes PAR [52], although a direct physical interaction in the absence of PARP-1 activity may also occur [38,40]. The possibility that p21 itself might be PARylated was investigated, but the extent of this modification appeared to be irrelevant (Fig 4B).

In our previous report, we suggested that p21 could regulate the release of PARP-1 from DNA damage sites, because chromatin-bound PARP-1 persisted longer in MNNG-treated p21^−/−^, than in p21^+/+^ human fibroblasts [40]. The present results clearly show that p21 regulates chromatin binding of the main PARP-1 form (113 kDa), while no useful information could be obtained from the recruitment of high MW bands, due to their limited amounts in the nuclear extracts. The unusual high basal levels of PARP-1 in the absence of DNA damage could explain why PAR levels were elevated in p21^−/−^ cells [40,54]. Thus, it is conceivable that persistently high levels of chromatin-bound PARP-1 and PAR, as observed in p21^−/−^ cells, could be detrimental to further DNA repair events [40].

Finally, it has to be taken into account that, after initial binding of PARP-1 to DNA damaged sites, a second wave of PARP-1 association to DNA occurs, which is dependent on PARP-1 activity [55]. According to this mechanism, p21 could provide help in the feedback-regulated PARylation of PARP-1, through the interaction with auto-modified PARP-1. This hypothesis is in agreement with our present evidence that p21 interaction with PARP-1 is favoured by PAR. After this second wave of PARP-1 recruitment and auto-modification, the decreased affinity for DNA may induce the subsequent PARP-1 release, necessary to avoid the inhibition of pol ß [48].

In conclusion, the above results indicate that not only initial PARP-1 binding to damaged DNA is favoured by p21, but also PARP-1 persistent binding to DNA damaged sites is modulated by p21 thanks to PAR binding. This may help in the feedback-regulated PARylation of PARP-1 [55], which ultimately results in its dissociation from DNA.

## Supporting Information

S1 Fig Olaparib significantly reduces the PAR synthesis in human cells. Human LF-1 fibroblasts grown on coverslips were treated for 30 min with 25 μM MNNG or pre-incubated with 10 μM Olaparib for 4 h before addition of MNNG. Cells were fixed in methanol/acetone (1:1, v/v) at 4°C [40]. After blocking with 1% (w/v) BSA in PBS-Tween 20 (0.2%, v/v) cells were incubated for 1 h with monoclonal antibody 10H to PAR, diluted 1:100. Secondary antibody was anti-mouse antibody conjugated with Dylight 488. DNA was counterstained with Hoechst 33258 (scale bar, 10 μm).
(PDF)

S2 Fig Olaparib reduces the number of cells showing co-localization of p21 with PARP-1. HeLa cells grown on coverslips were transfected with vectors for the expression of PARP-1-FL, or the AD domain, tagged with GST, and of p21-GFP. Forty-eight h later, cells were incubated with 100 μM MNNG for 30 min, after pre-incubation with 10 μM Olaparib for 4 h. Cells were then processed for hypotonic lysis in situ before fixation, as described [40]. Cells were stained with anti-GST antibody (1:100), and then labelled with a secondary antibody conjugated with Alexa 594 (red fluorescence); p21-GFP was detected by the green fluorescence. DNA was counterstained with Hoechst 33258 (Scale bar, 10 μm).
(PDF)
