# Supplementary figures and images for: p21CDKN1A Regulates the Binding of Poly(ADP-Ribose) Polymerase-1 to DNA Repair Intermediates
Source: PLoS One. 2016 Jan 5;11(1):e0146031. doi: 10.1371/journal.pone.0146031 (PMC4701469; doi:10.1371/journal.pone.0146031)

**S1 Fig**

**MNNG**

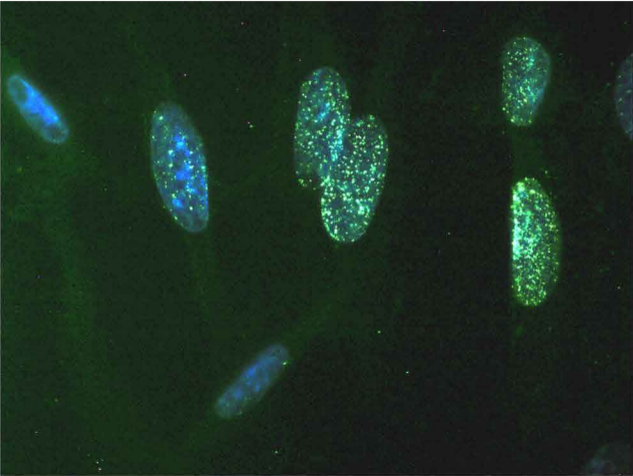

**MNNG + Olaparib**

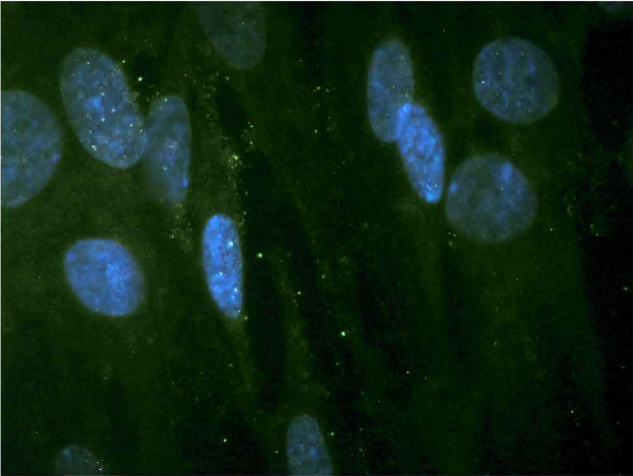

Supplement: S1 Fig — Human LF-1 fibroblasts grown on coverslips were treated for 30 min with 25 μM MNNG or pre-incubated with 10 μM Olaparib for 4 h before addition of MNNG. Cells were fixed in methanol/acetone (1:1, v/v) at 4°C [40]. After blocking with 1% (w/v) BSA in PBS-Tween 20 (0.2%, v/v) cells were incubated for 1 h with monoclonal antibody 10H to PAR, diluted 1:100. Secondary antibody was anti-mouse antibody conjugated with Dylight 488. DNA was counterstained with Hoechst 33258 (scale bar, 10 μm). (PDF) [file pone.0146031.s001.pdf]

**S2 Fig**

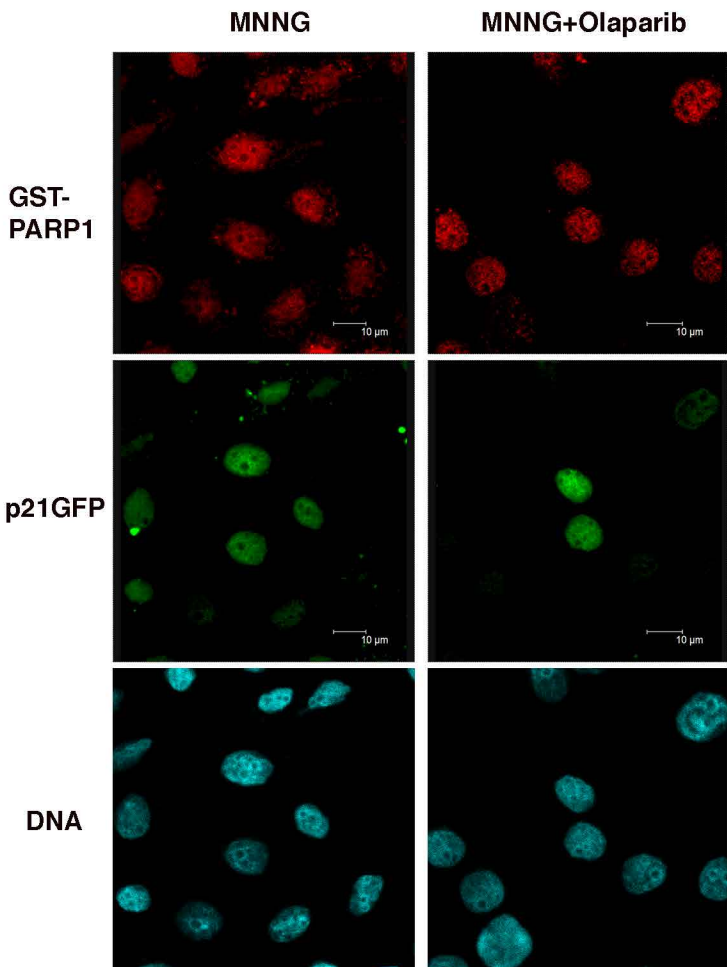

Supplement: S2 Fig — HeLa cells grown on coverslips were transfected with vectors for the expression of PARP-1-FL, or the AD domain, tagged with GST, and of p21-GFP. Forty-eight h later, cells were incubated with 100 μM MNNG for 30 min, after pre-incubation with 10 μM Olaparib for 4 h. Cells were then processed for hypotonic lysis in situ before fixation, as described [40]. Cells were stained with anti-GST antibody (1:100), and then labelled with a secondary antibody conjugated with Alexa 594 (red fluorescence); p21-GFP was detected by the green fluorescence. DNA was counterstained with Hoechst 33258 (Scale bar, 10 μm). (PDF) [file pone.0146031.s002.pdf]
